# Network inference from non-stationary spike trains

**DOI:** 10.1186/1471-2202-12-S1-P150

**Published:** 2011-07-18

**Authors:** Joanna Tyrcha, Yasser Roudi, John Hertz

**Affiliations:** 1 Department of Mathematical Statistics, Stockholm University, 106 91 Stockholm, Sweden; 2 Kavli Institute for Systems Neuroscience, NTNU, 7491 Trondheim, Norway; 3 Nordita, 106 91 Stockholm, Sweden; 4 Niels Bohr Institute, University of Copenhagen, 2100 Copenhagen Ø, Denmark

## 

Current approaches to the problem of inferring network connectivity from spike data [1,2] make a stationarity assumption, which is often not valid. Here we describe a method for inferring both the connectivity of a network in the presence of nonstationarity state and the time-dependent external drive that causes it.

Consider an experiment in which the network is subjected repeatedly to a potentially unknown external input (such as would arise from sensory stimulation). We assume the spikes to be binned in time and represented by a binary array: *S_i_*(*t*,*r*) = ±1, according to whether neuron *i* fires or not in time bin *t* of repetition *r* of the measurement. We fit these data to the simplest kind of binary stochastic model: At time step *t* of repetition *r*, each formal neuron receives a net input, *H_i_*(*t*,*r*) = *h_i_*(*t*) + ∑*_j_J_ij_S_j_*(*t*,*r*), and it takes the value +1 at the next step with a probability given by a logistic sigmoidal function 1/[1+exp(-2*H_i_*(*t*,*r*))] of *H_i_*(*t*,*r*). Maximizing the likelihood of the data leads to learning rules

for the model parameters -- the couplings *J_ij_* and external inputs *h_i_*(*t*). For weak coupling and/or densely connected networks, we have developed faster alternative algorithms [3]. These are based on expanding the learning rules around mean-field and TAP [4] equations for *m_i_*(*t*) = ‹*S_i_*(*t*,*r*)›*_r_*. (TAP equations are a generalization of the usual mean-field equations for highly connected random networks.)

**Figure 1:**
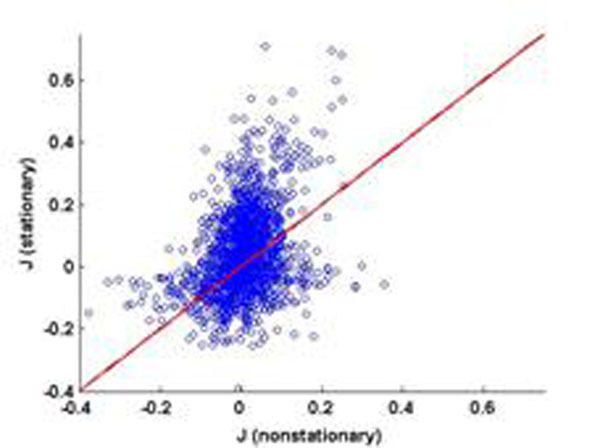
Couplings found assuming stationarity (y axis) plotted against coupling found not assuming stationarity (x axis) for data from salamander retina.

We have applied this method, as well as conventional ones assuming stationarity, to data sets from (1) the stochastic model itself, (2) a realistic computational model of a small cortical network, and (3) salamander retina under visual stimulation. In all three cases, we find that if we perform the reconstruction assuming stationarity, the algorithms effectively invent fictitious couplings to explain stimulus-induced correlations: The couplings in the network are systematically overestimated. The nonstationary treatment outlined above enables us, for sufficient data, to find both the correct (weaker) couplings and to extract the time-dependence of the external input. To illustrate this, figure 1 shows the *J_ij_*s found using the nonstationary algorithm plotted against those found using the stationary one, based on spike trains of 40 salamander retinal neurons stimulated by 120 repetitions of a 26.5-second clip from a film.. The mean *J_ij_* is reduced, from 0.0471 to -0.0028, and the large positive *J_ij_*s found assuming stationarity are reduced by a facto of 2-3 when nonstationarity is taken into account.
